# Intrasellar Chondroid Chordoma: A Case Report

**DOI:** 10.5402/2011/259392

**Published:** 2011-02-16

**Authors:** Renata M. Hirosawa, Antonio B. A. Santos, Mariana M. França, Viciany Erique Fabris, Ana Valéria B. Castro, Marco A. Zanini, Vania S. Nunes

**Affiliations:** ^1^Division of Endocrinology and Metabolism, Department of Internal Medicine, Faculty of Medicine of Botucatu, UNESP, 18618-970 Botucatu, SP, Brazil; ^2^Department of Pathology, Faculty of Medicine of Botucatu, UNESP, 18618-970 Botucatu, SP, Brazil; ^3^Division of Neurosurgery, Department of Neurology, Psychology and Psychiatry, Faculty of Medicine of Botucatu, UNESP, 18618-970 Botucatu, SP, Brazil

## Abstract

Chordomas are tumors derived from cells that are remnants of the notochord, particularly from its proximal and distal extremes, they are mainly midline and represent approximately 1% of all malignant bone tumors and 0.1 to 0.2% of intracranial neoplasms. Chordomas involving the sellar region are rare. Herein, we describe a 57-year-old male patient presenting with a history of retro-orbital headache, progressive loss of vision, and clinical features of hypopituitarism, for over 2 months. During evaluation, the CT scan revealed a large contrast-enhancing intrasellar tumor with a 3.6-cm largest diameter. The patient underwent transsphenoidal partial resection of the tumor, and histological examination was consistent with the diagnosis of chondroid chordoma. Although chordomas are rare, they may be considered to constitute a differential diagnostic of pituitary adenomas, especially if a calcified intrasellar tumor with bone erosion is diagnosed.

## 1. Introduction

Chordomas are midline slow-growing tumors that arise from proximal and distal extreme remnants of the notochord and represent 1% of all malignant bone tumors and 0.1 to 0.2% of intracranial neoplasms [1, 2]. Approximately 50% of them develop in the sacrococcygeal region, 35% in the spheno-occipital region, and 15% in the vertebrae [3]. They are locally invasive, may metastasize, and are characterized by local recurrence [4–6]. The incidence of endocranial chordomas presents a male/female ratio of 6 : 5 [7], and, at the skull base, the incidence is higher in younger patients (second to fifth decades of life) [2, 5, 8]. 

Three established categories of intracranial chordomas were described based on anatomical location and clinical features: sellar chordomas associated with chiasm compression and hypopituitarism; parasellar tumors characterized by oculomotor nerve palsy, optic tract compression, and hypopituitarism; tumors involving the clival region and presenting with bilateral sixth cranial nerve paresis and brainstem compression [9]. Chordomas involving the sellar region are rare, especially if they are largely or entirely intrasellar, and, clinically, they may resemble patients with pituitary adenoma [6, 10].

In this paper, we report a patient presenting with a largely intrasellar chordoma simulating a nonfunctioning pituitary adenoma.

## 2. Case Report

A 57-year-old man reported retro-orbital headache, diplopia, progressive loss of vision (predominantly on the right side), fatigue, nausea, sexual dysfunction, dry skin, sleepiness, and constipation over a period of two months. He also had a history of systemic arterial hypertension and was using a pacemaker due to chagasic heart disease. In his familial background, he reported diabetes mellitus, thyroid disease, a death caused by stroke, and no cases of tumors.

On physical examination, the patient presented postural hypotension and dry skin, any other alteration was not observed. Ophthalmological evaluation demonstrated a right-sided papillary defect, and visual field examination revealed a left temporal hemianopsia and a right/right-side blindness. 

**Figure 1:**
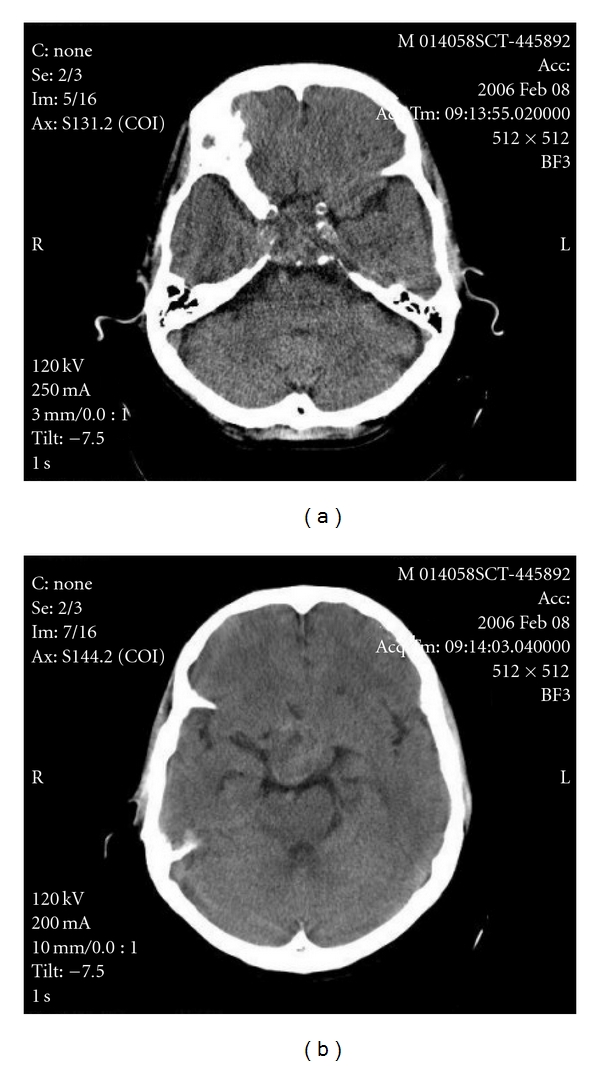
(a) and (b) CT shows a large heterogeneously contrast-enhanced intrasellar mass (3.6 cm in the largest diameter), with suprasellar extension, invasion of the sphenoid sinus, and erosion of the sellar floor.

**Figure 2:**
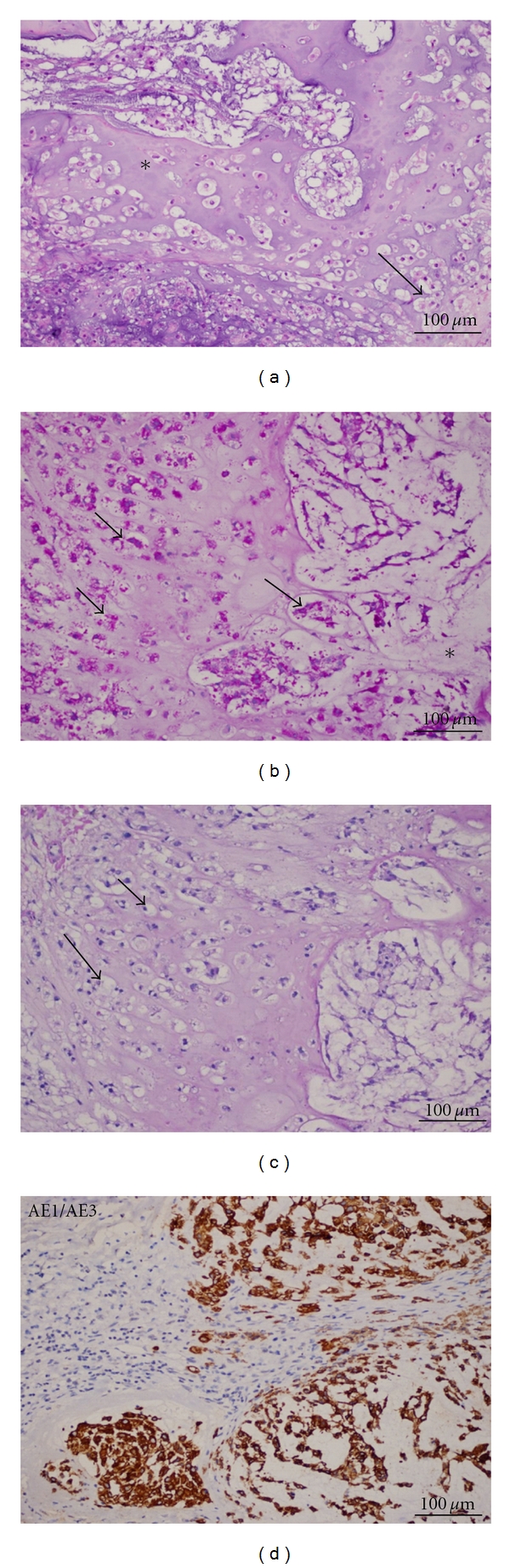
(a) Photomicrograph demonstrating the typical chordoma cells with large, pleomorphic nuclei and vacuolated cytoplasm (hematoxylin and eosin stain) forming sheets or lobular structures that are embedded in a mucoid stroma. Characteristic of chordomas chondroids are the formation of lobules of neoplastic tissue separated by fibrous stroma and areas of chondroid tissue (*), and vacuolated cell and lymphocytic infiltration. The tumor displays remarkable morphological variation, mainly based on the amount of interstitial matrix and vacuolated cells; these physaliphorous cells (arrow) with multivacuolated cytoplasm and sometimes pleomorphic nuclei are surrounded by mucinous extracellular matrix, some with chondroid aspect. Physaliphorous comes from the Greek word physalis (bubble). (b) PAS stain: the cytoplasm of many tumor cells is strongly PAS positive (arrows), highlighting them against mucoid matrix. The vacuoles, however, are usually negative. To define the nature of PAS positive material (glycogen), the technique was repeated in cuts, one of which was previously handled by diastase. (c) Neoplastic physaliphorous cells with arrows. (d) AE1/AE3: antibodies against keratins clearly show the ratio of neoplastic cells (dark brown) and interstitial tissue (in blue). The lobules of tumor tissue stand out, surrounded by strands of fibrous tissue.

**Figure 3:**
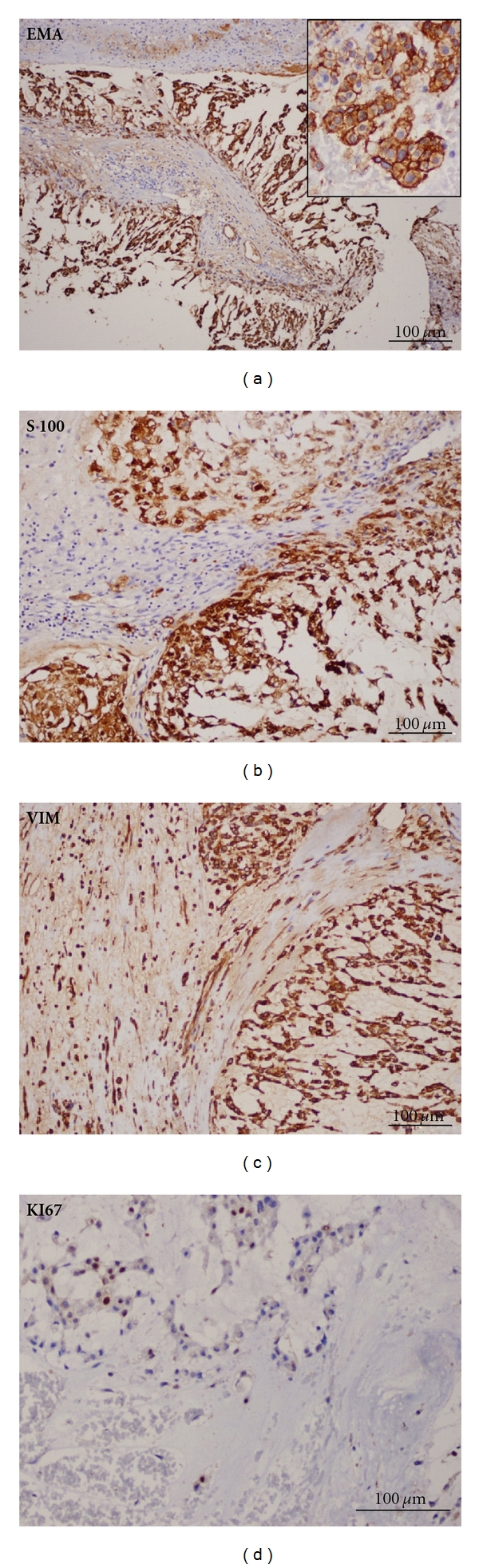
(a) EMA: epithelial membrane antigen positive cells of chordoma in papillary structure. Cytoplasmic pattern with enhanced cell membrane (insert). (b) S-100: strong nuclear and cytoplasmic positivity. (c) VIM. Cytoplasmic positivity. (d) KI67: positivity in a few scattered nuclei of neoplastic cells (less than 5%, low proliferation).

Biochemical evaluation revealed a slightly increased prolactin level (24.7 ng/mL, reference value (RV): 2.5–17); central hypothyroidism, and hypogonadism (TSH 0.23 *μ*IU/mL, RV: 0.4–4.0; free T4 0.51 ng/dL, RV: 0.8–1.9; FSH 1.45 mIU/mL, RV: 0.7–11.1; LH 0.23 mIU/mL, RV: 0.8–7.6; total testosterone <20 ng/dL, RV: 181–758); cortisol and IGF1 were normal (18 ug/dL, RV: 5–25; 237.3 ng/mL, RV: 78–258; resp.); GH 0.62 ng/mL (RV: 0.0–5.0); beta-chorionic gonadotropin (hCG), alpha-fetoprotein (FP), and carcinoembryonic antigen (CEA) were normal; diabetes insipidus investigation also was normal. It was not possible to realize a dynamic evaluation about the diagnosis of GH deficiency. The computed tomography (CT) scan revealed a large heterogeneous contrast-enhanced intrasellar mass (largest diameter: 3.6 cm), with suprasellar extension, invasion of the sphenoid sinus, and erosion of the sellar floor (Figures 1(a) and 1(b)). Magnetic resonance imaging (MRI) was not performed because of the presence of a pacemaker. Initially, the lesion was considered to be a nonfunctioning pituitary, and the patient was submitted to a transsphenoidal surgery. The tumor was not totally removed because of its tight adhesion to the pituitary stalk. Microcalcifications in the suprasellar and sellar portions were detected. Macroscopical findings showed multiple tan-to-red fragments. On light microscopic examination, the tumor was composed typically of a lobular pattern (Figures 2(d) and 3(b)). The lobes are separated by a framework of fibrous septa of variable thickness, which in sections appear as septa that run by the thin-walled vessels giving aspect of papillary structures (Figure 3(a)). It histologically showed a solid pattern or cordonal of anastomosing cell cords, with neoplastic cells embedded in a myxoid intercellular matrix with chondroid differentiation (Figure 2(a)). Tumor cells sometimes adopted a pattern of fine anastomosing cords or appeared in groups of varying sizes. Some cells showed epithelioid features with large and eosinophilic cytoplasm or clear cytoplasm, vacuolated cells contained a single vacuole that rejects the nuclei, creating an appearance of signet ring cell morphology, others show multiple vacuoles defining the physaliphorous cells. The contents of cytoplasmic vacuoles and intercellular mucoid substance are PAS positive and diastase resistant. Moreover, the cytoplasm of the neoplastic cells contains varying amounts of glycogen, PAS+, which is digested with diastase (Figures 2(b) and 2(c)). Physaliphorous cells contain multiple vacuoles surrounding the nucleus located in the center of the cell. The cellularity of the tumor was variable from field to field. Mitoses were very rare and atypical mitoses were not seen. Immunohistochemically, the neoplastic cells of chordoma were positive for cytokeratin AE1/AE3 (Figure 2(d)). They were characteristically positive chordoma cells for EMA (Figure 3(a)) and the protein S-100 (Figure 3(b)) and VIMENTIN (Figure 3(c)). The proliferation index was low as evidenced by weak nuclear positivity for Ki67 (Figure 3(d)). 

The patient received adequate hormone replacement before and following surgery. The postoperative evaluation showed hyponatremia associated to syndrome of inappropriate secretion of antidiuretic hormone (SIADH). The serum sodium normalized after fluid restriction. Four months later, the patient evolved with intense headache and significant worsening of the left-side vision. A frontal craniotomy was performed for total removal of the tumor. However, an aneurism of the anterior circulation was ruptured during the surgery. Therefore, the aneurism was clipped, but the tumor could not be removed. He stayed 19 days in the intensive care unit to receive pneumonia treatment, and he died two months later.

## 3. Discussion

We report the case of a patient presenting with a chondroid chordoma that had an aggressive clinical course.

Chordomas involving the sellar region are extremely rare. According to a recent review, only 22 patients have been described as possessing primarily intrasellar chordoma since 1960 and 43% of the patients were primarily diagnosed with a pituitary adenoma [3, 6, 9–21]. This rate of misdiagnosis might occur mainly because intrasellar chordomas mimic the clinical and imaging presentations of pituitary adenomas, as described in the present case and their higher frequency in relation to the chordomas [22]. 

The skull base chordomas tend to present osseous permeation, and have a high rate of recurrence [5, 6]. Their metastatic potential cannot be estimated by histological examination, except in cases of sarcomatous transformation [8, 23].

 The only known prognostic study published in 1993 estimated overall survival rates of 51% and 35% at 10 and 20 years, respectively [24]. 

The imaging method of choice for the pituitary gland is MRI, but coronal CT with contrast and thin slices may be obtained in patients who have contraindications to MRI. On unenhanced CT scan pituitary adenomas are isodense to adjacent brain parenchyma and show intense contrast enhancement after injection of contrast material. Bony erosions are well demonstrated in bone window images. A sellar chordoma CT scan may reveal a contrast-enhancing intrasellar mass with bony erosions and calcifications [25]. 

The immunohistologic profile of chordoma cells is reactivity to cytokeratins, vimentin, and epithelial membrane antigen (EMA) [26–33]. Approximately 50% of chordomas are reactive to S-100-protein [27]. Carcinoembryonic antigen in differential diagnosis has also been reported [30, 32–34]. Electron microscopy has contributed to the characterization of chordomas [27]. 

Transsphenoidal surgery (gross-total resection), followed by radiotherapy, has been the accepted treatment for sellar chordomas [5, 17, 35]. Unfortunately, in this patient, a radical excision was not possible because of adherence of the tumor to the pituitary stalk.

Although sellar chordomas are rare, they should be included in the differential diagnosis of tumors in the sellar region especially if a calcified tumor with bone erosion is revealed.
